# Population pharmacokinetics, target attainment and individualized dosing of isavuconazole in critically ill adults receiving extracorporeal membrane oxygenation

**DOI:** 10.1093/jacamr/dlag184

**Published:** 2026-08-25

**Authors:** Stefan Hatzl, Lisa Kriegl, Christina Geiger, Philipp Eller, Robert Krause

**Affiliations:** Intensive Care Unit, Department of Internal Medicine, Medical University of Graz, Graz, Austria; BioTechMed-Graz, Graz, Austria; BioTechMed-Graz, Graz, Austria; Division of Infectious Diseases, Department of Internal Medicine, Medical University of Graz, Auenbruggerplatz 15, Graz A-8036, Austria; Division of Infectious Diseases, Department of Internal Medicine, Medical University of Graz, Auenbruggerplatz 15, Graz A-8036, Austria; Intensive Care Unit, Department of Internal Medicine, Medical University of Graz, Graz, Austria; BioTechMed-Graz, Graz, Austria; Division of Infectious Diseases, Department of Internal Medicine, Medical University of Graz, Auenbruggerplatz 15, Graz A-8036, Austria

## Abstract

**Background and Objectives:**

Patients receiving extracorporeal membrane oxygenation (ECMO) for acute respiratory distress syndrome are at high risk of invasive pulmonary aspergillosis, but isavuconazole population pharmacokinetics and target attainment during ECMO remain incompletely characterized. To characterize isavuconazole population pharmacokinetics during ECMO, quantify between-patient variability and the probability of target attainment (PTA) and derive dosing implications.

**Patients and methods:**

Fifteen critically ill adults receiving ECMO and intravenous isavuconazole were studied prospectively, with 148 plasma concentrations measured over 168 h. First loading doses of 200, 300 or 400 mg reflected a stepwise change in institutional practice and were followed in all patients by 200 mg q8h × 5 and 200 mg q24h. Individual parameters were estimated by Bayesian forecasting against a literature-informed prior, and PTA was evaluated by simulating 5000 virtual subjects under five maintenance regimens.

**Results:**

Loading-phase exposure increased with the first dose (median AUC_0–24_ 14.2, 26.1 and 33.5 mg·h/L; *P* = 0.025) and converged at steady-state. Median Bayesian apparent clearance was 4.06 L/h, comparable with non-ECMO ICU populations, but between-patient variability was substantially wider (coefficient of variation 116% versus 38%; range 0.77–15.73 L/h). AUC_0_–τ  ≥ 60 mg·h/L was attained in four of seven patients with an evaluable steady-state profile, and simulation projected 41% attainment under standard maintenance. No regimen achieved >80% efficacy and >80% safety PTA simultaneously. Eight patients (53%) died.

**Conclusions:**

ECMO did not alter isavuconazole population clearance, so routine dose escalation is not warranted. Wide between-patient variability precludes any fixed regimen and favours therapeutic drug monitoring-guided individualized dosing.

## Introduction

Invasive pulmonary aspergillosis (IPA) has evolved from a disease occurring mainly in classically immunocompromised hosts into a major complication of critical illness. Influenza-associated and COVID-19–associated pulmonary aspergillosis have substantially expanded the at-risk population, and acute respiratory distress syndrome (ARDS) and post-cardiac-surgery complications are now recognized as potent risk factors for IPA, independent of classical EORTC-MSG criteria.^1–3^ Patients receiving extracorporeal membrane oxygenation (ECMO) as rescue therapy for refractory ARDS are therefore among those at highest risk.^4^ Isavuconazole is recommended as an alternative first-line treatment for invasive aspergillosis and offers more predictable pharmacokinetics and a better safety profile than voriconazole.^5–8^ ECMO might nevertheless complicate drug disposition, as the circuit alters protein binding and can sequester lipophilic drugs on polymeric membranes, a phenomenon well documented for voriconazole and more recently suggested for posaconazole.^9–11^

The pharmacokinetic profile of isavuconazole, characterized by a large volume of distribution, a terminal half-life of approximately 130 h, near-exclusive hepatic CYP3A4-mediated clearance with negligible renal elimination, linear pharmacokinetics across the therapeutic dose range and approximately 99% protein binding, supported the fixed dosing regimen used in the registration trials, where routine therapeutic drug monitoring (TDM) was not considered necessary.^5,12^ Data accumulated in intensive care populations have challenged that assumption. Apparent clearance in the ICU is roughly twice the pooled phase 1 and phase 3 estimate, approximately one-third of trough concentrations fall below 1 mg/L, and only about one-third of patients attain AUC_0_–τ  ≥ 60 mg·h/L on standard dosing.^13–16^ Hypoalbuminaemia with an increased unbound fraction, capillary leak, altered hepatic perfusion, inflammation-associated variability in CYP3A4 expression, obesity and renal replacement therapy have all been proposed as contributors.^13–15^

We previously showed that critically ill ECMO patients receiving standard isavuconazole loading (200 mg q8h × 6 doses) frequently failed to reach the proposed 1 mg/L target during the first 24 h and that an increased first loading dose of 400 mg achieved immediate and sustained target attainment without adverse effects.^7,17^ Neither pilot study characterized isavuconazole disposition, steady-state exposure or alternative maintenance regimens. It therefore remains unclear whether impaired target attainment during ECMO reflects circuit-related sequestration or the interindividual variability seen across critically ill populations generally. We characterized isavuconazole population pharmacokinetics in densely sampled patients receiving ECMO, quantified between-patient variability and evaluated the probability of target attainment (PTA) of alternative maintenance regimens by Monte Carlo simulation.

## Methods

### Ethics

The study was conducted in accordance with the Declaration of Helsinki and was reviewed and approved as non-interventional research by the institutional review board of the Medical University of Graz (33-062 ex 20/21). Written informed consent was obtained from all conscious participants; in unconscious patients, consent was deferred and obtained after recovery of decision-making capacity. Because participants were not assigned by the investigators to a therapeutic strategy and the only study-related procedure was withdrawal of additional blood from an arterial catheter already in place for routine care, the study did not meet the definition of a clinical trial under Directive 2001/20/EC or Regulation (EU) No 536/2014 and required neither authorization through the Clinical Trials Information System nor registration in a public trials registry.

### Study design and patients

We performed a single-centre, prospective, non-interventional pharmacokinetic study at the Medical University of Graz (Austria), extending the cohort previously reported.^7^ Between January 2021 and March 2023, consecutive critically ill adults receiving intravenous isavuconazole during ECMO support were included. The decision to start isavuconazole, the indication (mould-active prophylaxis or treatment of probable IPA) and the dose administered were made by the treating intensive care physicians according to institutional standard operating procedures and current guidelines, independently of and prior to inclusion.^1–3^ Demographic characteristics, comorbidities, indication, ECMO configuration, SOFA score and PaO_2_/FiO_2_ ratio were recorded at ECMO initiation; laboratory variables were recorded daily and are reported as the maximum observed during the 168-h sampling window. Exchanges of the ECMO circuit or membrane oxygenator, interacting co-medication, renal replacement therapy, adverse events and vital status were recorded prospectively (Supplementary Methods, available as Supplementary data at *JAC-AMR* Online).

### Dosing, ECMO circuits and sampling

The first isavuconazole loading dose used in our unit changed stepwise over the study period as an institutional practice adaptation, prompted by our earlier observation that the licensed loading regimen frequently fails to achieve the proposed target concentration during ECMO support.^7,17^ Patients treated in the earliest period received the licensed first dose of 200 mg (*n* = 7), followed by 300 mg (*n* = 3) and finally 400 mg (*n* = 5). All patients then received 200 mg q8h × 5 (six loading doses within 48 h) and 200 mg q24h from Day 3 onwards, each infusion administered over 60 min. Patients are reported by the first loading dose received in routine care; these strata are descriptive and were neither randomized nor allocated in advance. No patient received a moderate-to-strong CYP3A4 inducer or inhibitor during the sampling window. All ECMO circuits used a DP3 centrifugal pump (Medos Deltastream^®^) and a Novalung XLUNG Kit 230^®^ polymethylpentene membrane oxygenator (Xenios AG, Heilbronn, Germany). Blood was sampled from an indwelling arterial line at 2, 4, 8, 12, 16, 24, 48, 72, 96, 120, 144 and 168 h after the first dose, immediately before the next scheduled infusion where applicable; no sample was taken from a venous site or from the circuit. Total plasma isavuconazole concentrations were quantified by validated LC-MS/MS (Chromsystems, Gräfelfing, Germany) on a SCIEX QTRAP 4500 instrument as described previously.^17^

### Pharmacokinetic analysis and targets

Non-compartmental analysis was performed in R using the PKNCA package; steady-state AUC_0_–τ was estimated over the last fully sampled dosing interval (144–168 h) and apparent clearance as dose/AUC_0_–τ. Individual *post hoc* parameters were estimated by maximum *a posteriori* Bayesian estimation in mapbayr/mrgsolve against a literature-informed two-compartment prior with allometric scaling on body weight; a *de novo* SAEM fit in nlmixr2 served as a sensitivity analysis.^12,13^ PTA was evaluated by simulation of 5000 virtual subjects under five candidate maintenance regimens, each preceded by the standard 200 mg q8h × 6-dose loading, in two parallel scenarios: the literature-informed prior and log-normal distributions fitted to the cohort’s observed *post hoc* CL and V1. Three targets were applied *a priori*: a steady-state trough ≥1 mg/L for efficacy; AUC_0_–τ  ≥ 60 mg·h/L as an alternative exposure-based efficacy metric, corresponding to the 25th percentile of the exposure achieved by the licensed regimen in the adult phase 3 programme; and a steady-state trough <5 mg/L as a proposed safety threshold.^12,13,17,18^

Model parameters, simulation settings, target derivation, sampling-matrix considerations and outcome definitions are given in the Supplementary Methods.

### Statistics

Continuous variables are reported as median [IQR]; categorical as *n* (%). Comparisons across the three loading-dose strata used the Kruskal–Wallis test, with *post hoc* Mann–Whitney *U* tests reported as exploratory; categorical variables were compared by Fisher’s exact or χ2 test. Bayesian *post hoc* clearance was additionally compared between veno-venous and other ECMO configurations (Mann–Whitney *U*). Survival was analysed from ECMO initiation by Kaplan–Meier estimation with log-rank comparison and univariable Cox regression; multivariable models were not fitted given the modest event count.^19^ A two-sided α = 0.05 was used without correction for multiple comparisons, and all *P* values reported in the text correspond to those given in Table 2.

## Results

### Study cohort

Fifteen critically ill adults receiving ECMO and intravenous isavuconazole were included. Seven patients (47%) received 200 mg, three (20%) 300 mg and five (33%) 400 mg as the first loading dose. Twelve patients (80%) received veno-venous ECMO, two (13%) veno-arterial and one (7%) veno-arterio-venous ECMO. At ECMO initiation, the cohort comprised severely ill patients, with a median PaO_2_/FiO_2_ of 76 mmHg [IQR 65–87] and SOFA score 8.5 [8–12]. Twelve patients (80%) received isavuconazole as antifungal prophylaxis and three (20%) for treatment of probable invasive pulmonary aspergillosis. Two patients (13%) received continuous renal replacement therapy during the sampling window, and no exchange of the ECMO circuit or membrane oxygenator took place in any patient. Cohort characteristics are presented in Table 1.

**Table 1.** Clinical description of the overall cohort by isavuconazole loading-dose group

Variable	Overall (*N* = 15)	First dose 200 mg (*n* = 7)	First dose 300 mg (*n* = 3)	First dose 400 mg (*n* = 5)	*P*
***Demographic variables***					
Age (years)	62 [47–64]	60 [45–64]	67 [31–72]	63 [62–63]	0.527
Females, *n* (%)	7 (47%)	3 (43%)	1 (33%)	3 (60%)	0.736
Body weight (kg)	84.2 [62.1–100.1]	96.1 [82.4–103.9]	65.1 [62.5–65.1]	64.6 [57.5–92.2]	0.054
BMI (kg/m^2^)	26.0 [22.0–31.4]	29.8 [26.9–35.2]	22.0 [18.5–24.8]	22.0 [21.1–27.5]	0.126
***Coexisting conditions***					
No coexisting conditions	9 (60%)	3 (43%)	2 (66%)	4 (80%)	0.418
Cardiovascular disease	4 (27%)	2 (29%)	1 (33%)	1 (20%)	0.907
Diabetes mellitus	3 (20%)	0 (0%)	2 (66%)	1 (20%)	0.054
Haematological neoplasm	1 (7%)	0 (0%)	0 (0%)	1 (20%)	0.343
Immunocompromised	4 (27%)	1 (15%)	1 (33%)	2 (40%)	0.585
Respiratory disease	2 (14%)	1 (15%)	0 (0%)	1 (20%)	0.719
***Indication and ICU characteristics***					
Antifungal prophylaxis, *n* (%)	12 (80%)	7 (100%)	1 (33%)	4 (80%)	0.025
Treatment of probable IPA, *n* (%)	3 (20%)	0 (0%)	2 (66%)	1 (20%)	0.025
ECMO mode					0.453
Veno-venous	12 (80%)	6 (86%)	2 (66%)	4 (80%)	
Veno-arterial	2 (14%)	1 (14%)	1 (33%)	0 (0%)	
Veno-arterial-venous	1 (7%)	0 (0%)	0 (0%)	1 (20%)	
paO_2_/FiO_2_ (mmHg)	76 [65–87]	78 [68–87]	76 [75–89]	61 [58–78]	0.429
SOFA score	8.5 [8–12]	8 [8–10]	12 [11–20]	8 [7–9]	0.162
Acute kidney injury (KDIGO)					0.840
AKI stage 1	2 (14%)	1 (14%)	1 (33%)	0 (0%)	
AKI stage 2	2 (14%)	1 (14%)	0 (0%)	1 (20%)	
AKI stage 3	2 (14%)	1 (14%)	0 (0%)	1 (20%)	
Continuous renal replacement therapy	2 (13%)	1 (14%)	0 (0%)	1 (20%)	1.000
***Laboratory values***					
Creatinine (mg/dL), max	0.77 [0.53–0.98]	0.73 [0.53–0.98]	0.98 [0.47–1.19]	0.77 [0.53–0.78]	0.699
Bilirubin (mg/dL), max	0.73 [0.45–0.89]	0.69 [0.36–0.87]	0.78 [0.15–0.96]	0.75 [0.70–0.99]	0.499
AST (U/L), max	38 [24–58]	38 [25–58]	26 [24–96]	43 [24–48]	0.989
ALT (U/L), max	34 [17–45]	45 [15–47]	36 [24–39]	25 [17–28]	0.334
***Outcomes***					
ICU length of stay (days)	19 [10–23]	22 [19–39]	18 [4–41]	10 [9–12]	0.065
ECMO duration (days)	13 [7–15]	15 [13–19]	8 [4–15]	9 [7–11]	0.094
Deceased at data cut-off	8 (53%)	3 (43%)	2 (66%)	3 (60%)	0.736
Median survival from ECMO start (days)	20 [10–115]	22 [10–502]	15 [4–18]	10 [9–12]	0.412

Continuous variables are presented as median [IQR]; categorical variables as *n* (%). *P* values represent comparisons across the three loading-dose groups (Kruskal–Wallis for continuous variables; Fisher’s exact or χ2 for categorical, as appropriate).

Laboratory values reflect cohort-typical maxima abstracted from the medical record. Per protocol, no patient received concomitant moderate-to-strong CYP3A4 modulators during the sampling window.

AKI, acute kidney injury; KDIGO, Kidney Disease Improving Global Outcomes; IPA, invasive pulmonary aspergillosis; SOFA, sequential organ failure assessment; PaO_2_/FiO_2_, partial pressure of oxygen/fraction of inspired oxygen; AST, aspartate aminotransferase; ALT, alanine aminotransferase.

### Loading-phase exposure increases proportionally with the first loading dose

**Figure 1.**
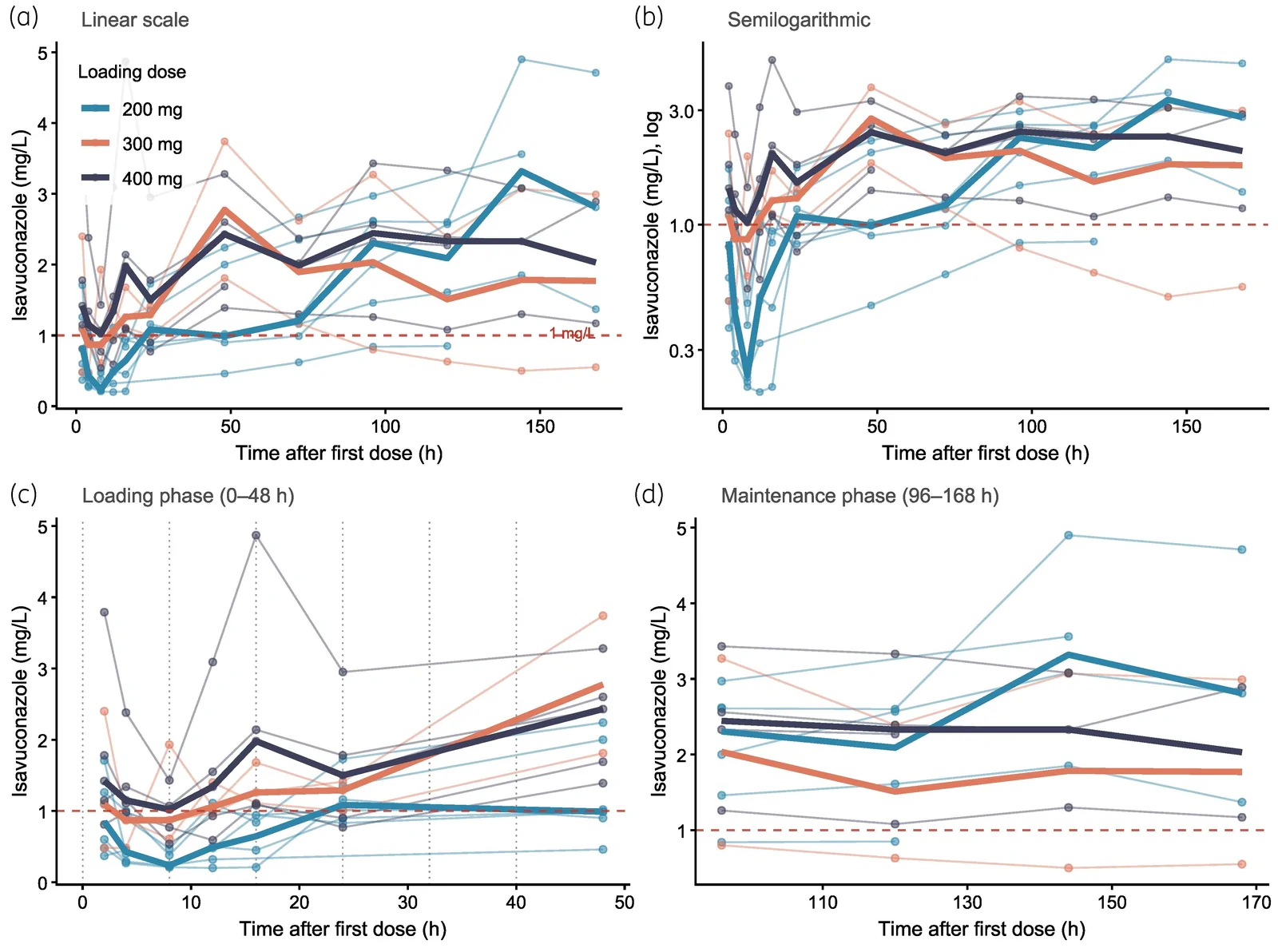
Isavuconazole plasma concentrations during ECMO support (*n* = 15). (a) Concentration–time profiles, linear scale, with individual trajectories (thin lines) and group medians (thick lines) for the 200, 300 and 400 mg loading-dose groups. (b) Same data on a semilogarithmic axis. (c) Loading-phase detail (0–48 h) showing dose-proportional increase in early exposure with each loading-dose escalation; vertical dotted lines indicate scheduled q8h doses. (d) Steady-state phase (96–168 h), showing convergence of concentrations across loading-dose groups under uniform 200 mg q24h maintenance. Red dashed line: efficacy threshold of 1 mg/L.

Loading-phase exposure rose progressively with each escalation of the first dose. Median AUC_0–24_ was 14.2 [9.8–19.3] mg·h/L for the 200 mg group, 26.1 [25.7–27.9] mg·h/L for the 300 mg group and 33.5 [23.0–36.8] mg·h/L for the 400 mg group (*P* = 0.025; pairwise 200 versus 400 mg *P* = 0.030). Median Cmax and the lowest concentration observed during the interval showed the same trend without reaching significance (1.26, 1.93 and 1.98 mg/L, *P* = 0.052; 0.24, 0.87 and 1.02 mg/L, *P* = 0.052). All five patients receiving 400 mg as the first dose reached the 1 mg/L threshold within 16 h, four of them within 2 h. Transient attainment within 24 h was nevertheless common in every stratum (5/7, 3/3 and 5/5 for 200, 300 and 400 mg). At the 24 h trough, the first point at which attainment can be regarded as sustained, the proportions were four of six evaluable patients (200 mg; one had no 24 h sample), three of three (300 mg) and three of five (400 mg), two of the latter having fallen back to 0.90 and 0.77 mg/L. Threshold attainment during loading therefore did not separate the strata, whereas AUC_0–24_ did (Table S4). No concentration exceeded 5 mg/L during loading (Figure 1, Table 2).

**Table 2.** Pharmacokinetic parameters and target attainment, stratified by isavuconazole loading-dose group

Parameter	Overall (*N* = 15)	First dose 200 mg (*n* = 7)	First dose 300 mg (*n* = 3)	First dose 400 mg (*n* = 5)	*P*
***Loading phase (0–24 h after first dose)***					
Cmax (mg/L)	1.71 [1.21–1.96]	1.26 [1.03–1.53]	1.93 [1.67–2.17]	1.98 [1.78–2.14]	0.052
Trough min (mg/L)^1^	0.61 [0.31–1.04]	0.24 [0.23–0.42]	0.87 [0.74–1.08]	1.02 [0.77–1.07]	0.052
AUC_0–24_ (mg·h/L)^2^	23.1 [17.7–28.7]	14.2 [9.8–19.3]	26.1 [25.7–27.9]	33.5 [23.0–36.8]	0.025
AUC_0–48_ (mg·h/L)	54.0 [37.7–76.7]	41.5 [34.0–49.1]	59.8 [44.7–73.5]	82.7 [54.0–87.3]	0.098
***Steady-state (144–168 h)***					
Cmax (mg/L)^3^	3.07 [1.85–3.08]	3.32 [2.77–3.90]	1.81 [1.18–2.44]	2.89 [2.10–2.99]	0.250
Ctrough (mg/L)^4^	2.45 [1.22–2.97]	2.62 [1.18–3.19]	1.77 [1.16–2.38]	2.27 [1.69–2.89]	0.860
AUC_0_–τ (mg·h/L)^5^	62.6 [34.1–71.7]	70.7 [54.7–93.0]	42.7 [27.6–57.7]	46.1 [37.9–54.4]	0.555
Css,avg (mg/L)^5^	2.61 [1.42–2.99]	2.95 [2.28–3.88]	1.78 [1.15–2.40]	1.92 [1.58–2.27]	0.555
Apparent CL (L/h)^5^	3.19 [2.79–5.96]	2.83 [2.28–4.00]	9.31 [6.03–12.59]	4.97 [4.08–5.86]	0.555
***Bayesian individual post hoc estimates (n*** **=** ***15 evaluable)***					
Apparent CL (L/h)	4.06 [2.14–10.85]	2.48 [1.72–9.26]	5.42 [3.72–10.57]	4.09 [2.32–7.99]	0.840
V1 (L)	218 [135–288]	162 [141–366]	135 [101–294]	270 [218–411]	0.410
***Target attainment, n (%)***					
Trough ≥1 mg/L at SS^4^	11/14 (79%)	5/7 (71%)	1/2 (50%)	5/5 (100%)	0.420
AUC_0_–τ ≥ 60 mg·h/L at SS^5^	4/7 (57%)	2/3 (67%)	1/2 (50%)	1/2 (50%)	1.000
All concentrations <5 mg/L throughout (safety)^6^	15/15 (100%)	7/7 (100%)	3/3 (100%)	5/5 (100%)	1.000

Isavuconazole pharmacokinetic parameters during the loading phase, at steady-state and from Bayesian individual estimation, stratified by loading dose (200, 300 and 400 mg). Loading-phase exposure scaled with dose (median AUC_0–24_ 14.2, 26.1 and 33.5 mg·h/L; *P* = 0.025) but converged at steady-state.

AUC ≥60 was attained in four of seven evaluable patients; trough ≥1 mg/L in 11 of 14; no concentration in any patient exceeded the 5 mg/L safety ceiling; Cmax, maximum concentration; Ctrough, trough concentration immediately before next dose; AUC_0–24_, area under the concentration–time curve from 0 to 24 h; AUC_0_–τ, area under the concentration–time curve over one dosing interval at steady-state; Css,avg, average steady-state concentration; CL, apparent clearance; V1, volume of distribution of the central compartment; SS, steady-state; TDM, therapeutic drug monitoring.

### Steady-state exposure converges across loading-dose groups

By the steady-state interval (144–168 h), exposure was determined predominantly by the maintenance dose, which was uniform (200 mg q24h) in all patients. Median AUC_0_–τ was 70.7, 42.7 and 46.1 mg·h/L for the 200, 300 and 400 mg strata (*P* = 0.555), median steady-state trough concentrations 2.62, 1.77 and 2.27 mg/L (*P* = 0.860), and median apparent clearance derived from the steady-state interval 2.83, 9.31 and 4.97 L/h (*P* = 0.555); interquartile ranges are given in Table 2 (Figure 1).

Steady-state exposure was thus numerically lower in the strata that had received the higher first loading dose. This cannot be a carry-over effect of the loading dose, which contributes negligibly to exposure 5 to 7 days later. It is explained by the small number of patients with a completely sampled final dosing interval (three, two and two, respectively) together with the wide variability in clearance described below: within this subset, median apparent clearance differed more than 3-fold, whereas the Bayesian *post hoc* estimates in all 15 patients differed far less (2.48, 5.42 and 4.09 L/h; *P* = 0.840). No steady-state comparison approached significance, and we therefore do not interpret these data as indicating that a higher first loading dose lowers steady-state exposure.

### ECMO does not alter population-level isavuconazole clearance

**Figure 2.**
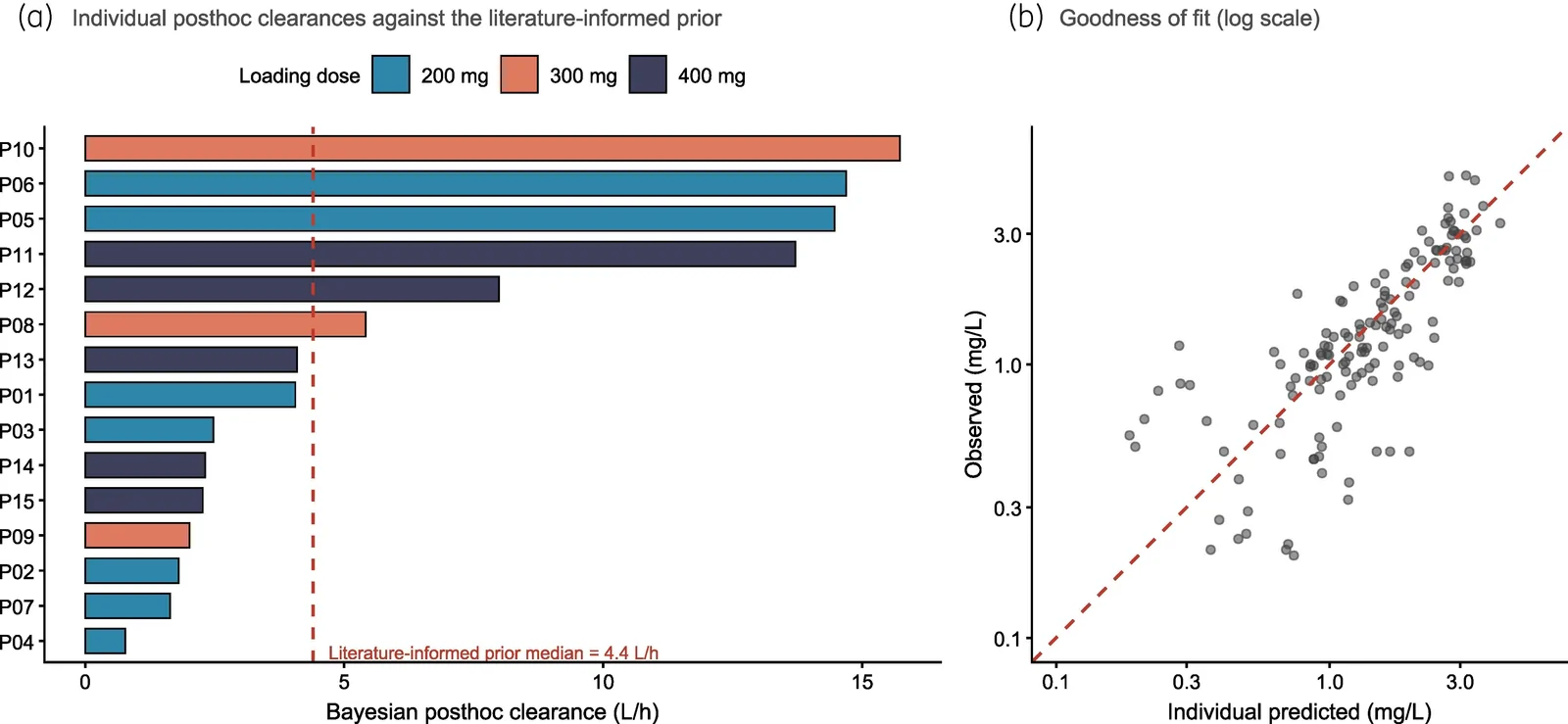
Bayesian individual *post hoc* clearance estimates against the literature-informed prior. (a) Distribution of individual clearance values (log-normal CV 116%); the dashed horizontal line indicates the prior population value of 4.4 L/h. (b) Goodness-of-fit plot of observed versus individual predicted concentrations on a log-log scale; the red dashed line is the line of identity. The absence of systematic bias supports the appropriateness of the prior for the ECMO cohort and indicates that ECMO does not alter isavuconazole disposition at the population level.

Individual Bayesian *post hoc* apparent clearance varied more than 20-fold across the cohort, from 0.77 to 15.73 L/h (Figure 2a; individual profiles in Figure S2). The cohort median was 4.06 [IQR 2.14–10.85] L/h, broadly within the range reported across published populations, modestly higher than the pooled phase 1+3 SECURE estimate of 2.36 L/h and consistent with the upward shift observed in critically ill cohorts.^5,13,20^ Between-patient variability was, however, substantially wider than in published populations, with a log-normal coefficient of variation of 116% on clearance versus 38%–62%.^13,14,20^ Despite this heterogeneity, the literature-informed prior described the observed data without systematic bias across the full concentration range of 0.2–5 mg/L (Figure 2b; full goodness-of-fit diagnostics in Figure S1), indicating that ECMO support does not shift isavuconazole disposition at the population level.

Four patients (27%) had clearance exceeding 10 L/h despite weights, comorbidities and co-medication similar to the rest of the cohort. Bayesian *post hoc* clearance did not differ appreciably between ECMO configurations (median 3.27 [IQR 1.96–9.42] L/h for veno-venous support, 10.06 L/h for veno-arterial and 2.32 L/h for the single patient on veno-arterio-venous support; *P* = 0.45 for veno-venous versus other), and three of the four patients with a clearance above 10 L/h were supported with veno-venous ECMO (Table S1); with two and one patients in the non-veno-venous strata, this comparison is descriptive only. A *de novo* SAEM population fit yielded CL = 2.4 L/h (95% CI 1.0–5.6) with very wide uncertainty and severe shrinkage on volume parameters, reflecting the limited sample size (Table S1, Figure S3).

### Target attainment with current standard dosing is suboptimal

**Figure 3.**
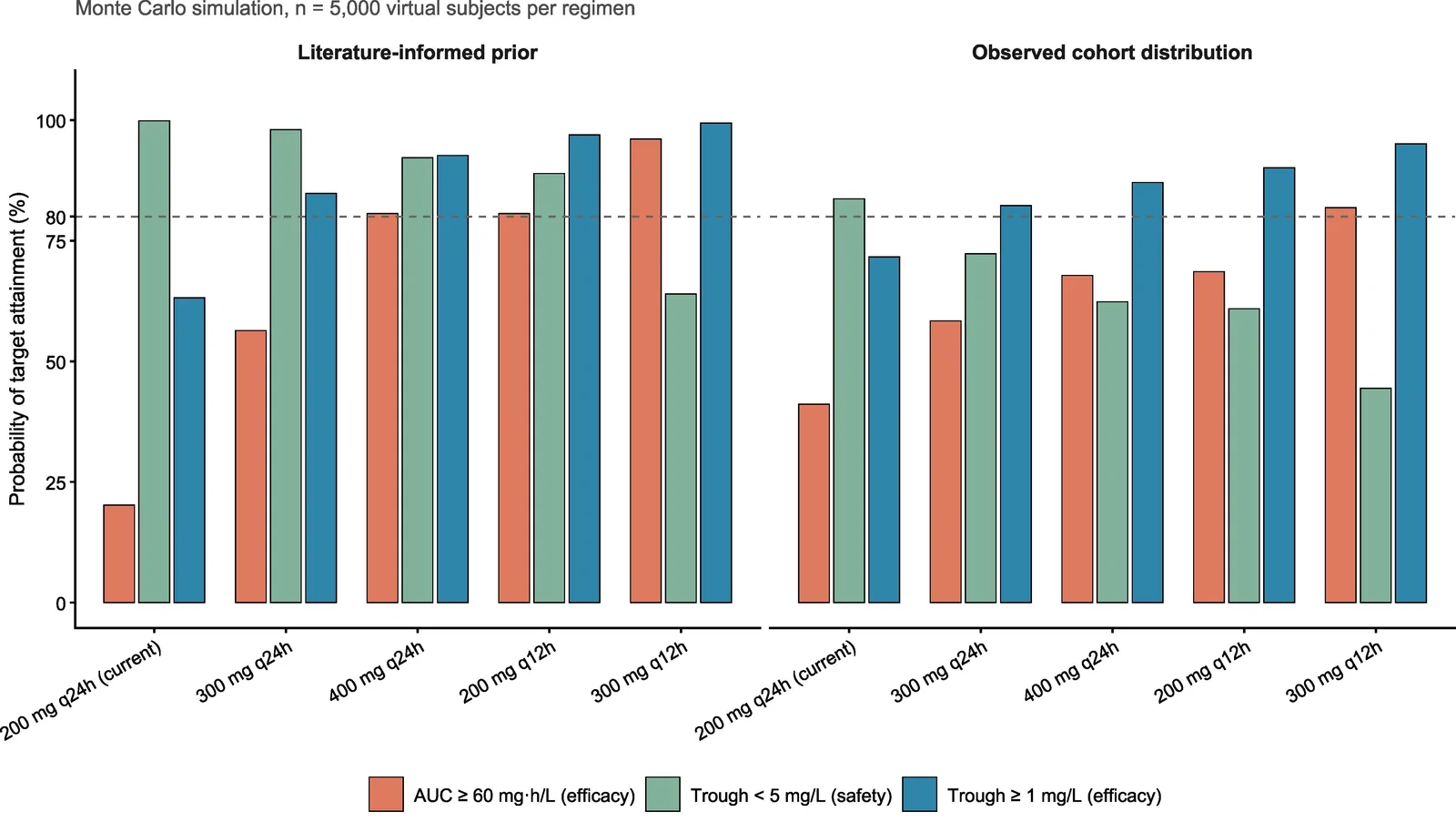
PTA for alternative maintenance dosing regimens, from Monte Carlo simulation of 5000 virtual subjects. Left panel: simulation using literature-informed prior. Right panel: simulation using the cohort’s observed log-normal clearance and central volume distribution (observed-cohort scenario; fitted projection for an ECMO-like population). Bars show PTA for AUC_0–24_  ≥ 60 mg·h/L (efficacy), trough ≥1 mg/L (efficacy) and trough <5 mg/L (safety). The dashed horizontal line is the 80% PTA benchmark. No regimen simultaneously achieves >80% efficacy and >80% safety PTA in the observed-cohort scenario.

With current standard maintenance dosing, four of seven patients with an evaluable steady-state profile (57%) achieved AUC_0_–τ  ≥ 60 mg·h/L, and 11 of 14 patients with a measurable steady-state trough (79%) achieved trough ≥1 mg/L (Table 2). Forward Monte Carlo simulation using the cohort’s observed CL and V1 distributions projected an AUC PTA of 41.1% under 200 mg q24h dosing, of the same order as observed. Higher maintenance doses raised projected efficacy PTA (58.4% for 300 mg q24h and 67.8% for 400 mg q24h); only 300 mg q12h approached the 80% benchmark (81.8%), at a safety PTA of just 44.4%. Across all five regimens, no fixed dose simultaneously achieved >80% efficacy and >80% safety PTA, with the joint PTA ranging from 24.8% to 30.7% (Figure 3; full outputs in Table S2). Equivalent simulations using the literature-informed prior yielded systematically higher PTA estimates, reflecting the prior’s narrower variability assumption (Figure 3, left panel).

### Safety, hepatic tolerance and breakthrough infection

No concentration exceeded the proposed 5 mg/L safety threshold at any time point in any patient; the highest observed anywhere in the dataset was 4.90 mg/L. Liver function tests remained largely unremarkable (maximum aspartate aminotransferase 38 [24–58] U/L, alanine aminotransferase 34 [17–45] U/L and total bilirubin 0.73 [0.45–0.89] mg/dL), without significant differences across strata (Table 1). No patient fulfilled criteria for drug-induced liver injury, and no patient required dose reduction, interruption or discontinuation of isavuconazole because of a suspected adverse drug reaction, either during loading or at steady-state. Among the 12 patients receiving mould-active prophylaxis, no breakthrough invasive fungal infection was observed during isavuconazole exposure or within 7 days of its discontinuation.

### Mortality and exposure–response

**Figure 4.**
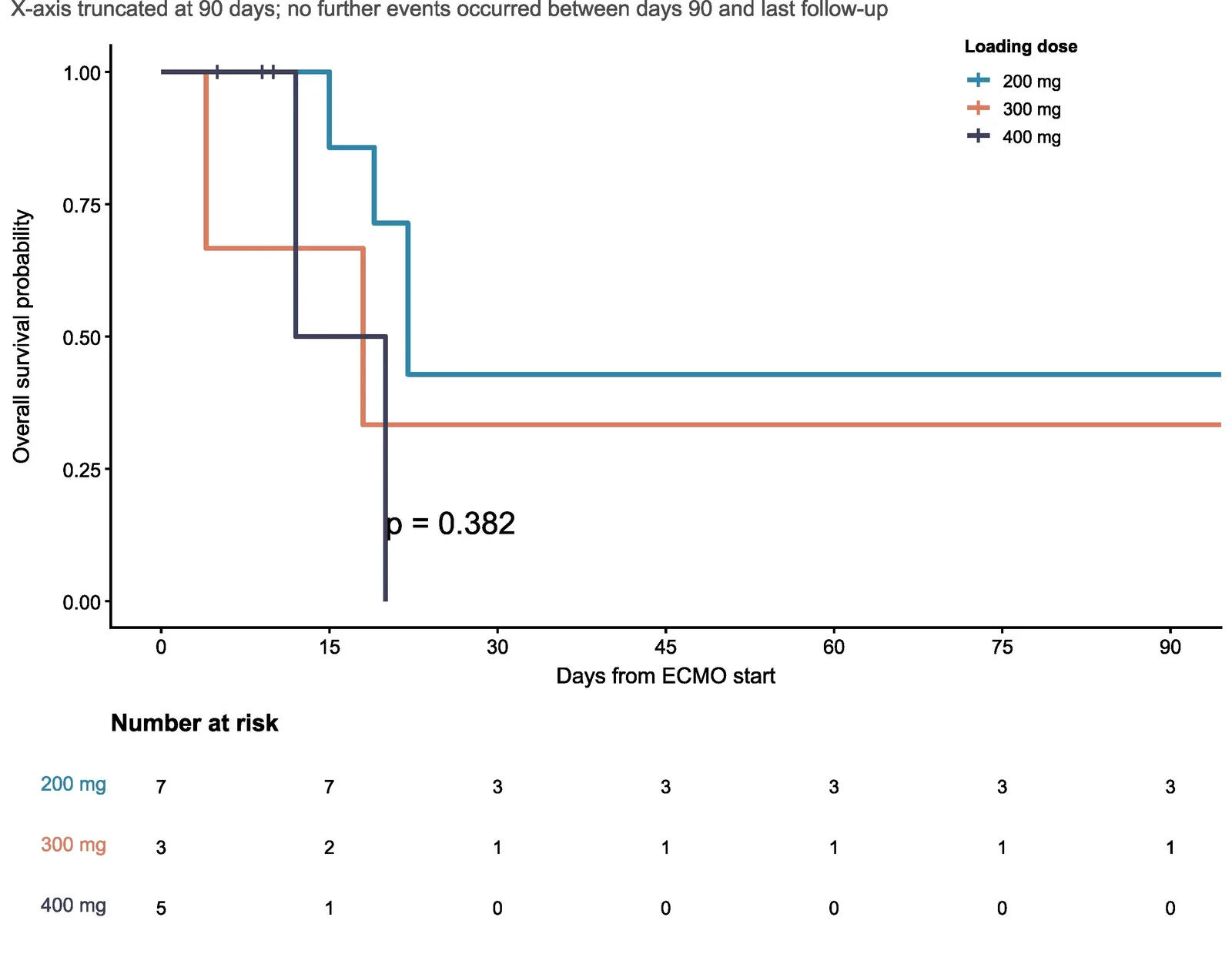
Kaplan–Meier survival from the date of ECMO initiation, stratified by isavuconazole loading-dose group (200, 300 and 400 mg as the first dose). Risk table beneath. X-axis truncated at 90 days; no further events occurred between Day 90 and last follow-up. Log-rank *P* value annotated. Survival results are presented as supportive cohort description; the analysis is severely underpowered (8 events in 15 patients) for detecting exposure–response relationships. Survival stratified by exposure metrics is shown in Figure S4.

Median follow-up of survivors was 115 days. Eight of 15 patients (53%) had died at data cut-off, with median time to death of 18 days. All three patients treated for probable invasive pulmonary aspergillosis died, compared with 5/12 (42%) receiving prophylaxis; these three had received a first dose of 300 mg (*n* = 2) or 400 mg (*n* = 1). Survival did not differ significantly across loading-dose groups (*P* = 0.382; Figure 4), by attainment of AUC_0_–τ  ≥ 60 mg·h/L at steady-state (*P* = 0.321; Figure S4A) or by tertile of Bayesian *post hoc* clearance (*P* = 0.653; Figure S4B). In univariable Cox regression, mortality was associated with the indication for isavuconazole, i.e. treatment versus prophylaxis (HR 14.99, 95% CI 1.53–147.04, *P* = 0.020) and increasing age (HR 5.87 per 10 years, 95% CI 1.08–31.92, *P* = 0.040); loading dose, AUC target attainment, SOFA score and Bayesian clearance were not significantly associated with outcome (Table S3).

## Discussion

In this prospective study of 15 critically ill patients receiving ECMO, isavuconazole population pharmacokinetics did not differ systematically from those reported in non-ECMO ICU cohorts: median apparent clearance lay within the published range, and an ICU prior fitted our data without systematic bias.^13,14^ The likely explanation is the high protein binding of isavuconazole (approximately 99%), which appears to protect it from the polymeric-membrane sequestration demonstrated for voriconazole and is reflected in our previous finding of comparable pre- and post-oxygenator concentrations.^10,11,17^ Routine pre-emptive escalation of the maintenance dose for ECMO support alone is therefore not justified.

Between-patient variability, by contrast, was substantial. Apparent clearance ranged more than 20-fold (0.77–15.73 L/h), and the log-normal coefficient of variation of 116% substantially exceeded the 38% of the literature-informed prior, although, derived from 15 *post hoc* estimates, it is imprecise and may partly reflect parameter uncertainty.^13^ Four patients had a clearance above 10 L/h without identifiable covariates that would allow prospective recognition. This variability, rather than ECMO itself, is the proximate cause of target attainment failure: AUC_0_–τ  ≥ 60 mg·h/L was documented in only four of seven patients with an evaluable steady-state profile, and simulation using the cohort’s own parameter distributions projected attainment in 41% of an ECMO-like population, closely mirroring the 35.8% attainment and the high proportion of subtherapeutic troughs reported in non-ECMO ICU cohorts.^13,14^ The clinical consequence is that no fixed regimen satisfied both targets: raising maintenance from 200 to 400 mg daily lifted efficacy PTA from 41% to only 68% while pushing more than one-third of patients above the 5 mg/L threshold, and the 80% benchmark was reached only with 300 mg q12h, at which fewer than half remained within safe concentrations.

These findings support a TDM-guided dosing strategy rather than the universal empirical dose escalation we previously suggested: because population clearance was not increased, the case for escalating the dose in every patient is weak, whereas the case for measuring it in every patient is strong. A higher first loading dose may be considered to shorten the interval to the target concentration, since loading-phase exposure increased proportionally with the first dose and no concentration approached the safety threshold during loading.^7,13^ The present cohort tempers our earlier report of sustained attainment with a 400 mg first dose: all five such patients crossed 1 mg/L within 16 h, but two fell below it again by the 24 h trough, and attainment at 24 h did not differ across strata. A higher first dose therefore accelerates early exposure without guaranteeing that the threshold is held. This observation rests on five patients; the optimal loading strategy and first dose remain to be established, and the licensed loading regimen remains an acceptable alternative until larger prospective data are available. Maintenance should remain at 200 mg q24h and be adjusted on a trough concentration measured between Days 5 and 7 immediately before the next dose: below 1 mg/L, escalation to 300 mg q24h with a repeat trough after 3–5 days; above 5 mg/L, assessment for hepatic and gastrointestinal toxicity and consideration of dose reduction. Because none of the available covariates identified the four high-clearance patients, we consider TDM appropriate in all patients receiving isavuconazole during ECMO and indispensable in those treated for invasive mould disease rather than prophylaxis, in those with isolates of elevated MIC or progression on therapy, in those with marked hypoalbuminaemia, hepatic dysfunction or interacting co-medication, and at the extremes of body weight. The same principle has been proposed for voriconazole, posaconazole and isavuconazole in non-ECMO ICU populations, and our data extend it to patients receiving ECMO.^11,13,15–17,21,22^

The relevance of optimizing isavuconazole exposure during ECMO is amplified by the evolving epidemiology of IPA, now a major complication of severe ARDS, including its viral aetiologies, and of cardiac surgery.^2,3,23^ Patients in whom veno-venous ECMO is initiated for refractory ARDS overlap substantially with this newly recognized at-risk population, in whom subtherapeutic antifungal exposure may matter.^2,24^

Several limitations warrant acknowledgement. The cohort is small (*n* = 15) and from a single centre, limiting precision and generalizability, although it remains the largest densely sampled isavuconazole pharmacokinetic dataset in patients receiving ECMO. The Bayesian analysis depends on a prior derived from a non-ECMO population, which may not capture all features of ECMO disposition.^13^ Free concentrations were not measured, and our analyses assume a constant unbound fraction, a simplification given that critical illness and hypoalbuminaemia may shift binding. All samples were drawn from an arterial line, whereas the applied thresholds derive from venous sampling; arterio-venous differences should be negligible at trough and during maintenance, when all target-defining concentrations were assessed, but loading-phase peaks may have been modestly higher than venous values. The cohort was dominated by veno-venous support (12 of 15 patients), so a modality effect cannot be excluded. The exposure targets are derived from treatment cohorts and have not been validated for prophylaxis, the indication in 12 of 15 patients, so attainment in that setting should be read as a pharmacokinetic benchmark rather than a validated clinical threshold. Only five patients received a 400 mg first dose, so all statements on the loading strategy are exploratory. Finally, the survival analysis is severely underpowered (eight events) and cannot exclude a clinically important exposure–response relationship; the association of indication with mortality reflects the severity of probable IPA rather than an exposure–outcome link and is confounded because all seven patients given 200 mg received prophylaxis, whereas the three treated for probable IPA received 300 mg (*n* = 2) or 400 mg (*n* = 1). A multicentre TDM-guided dosing trial powered for clinical outcomes and incorporating free-drug measurement is the logical next step.

In summary, isavuconazole population pharmacokinetics in critically ill patients receiving ECMO do not differ systematically from non-ECMO ICU populations, arguing against clinically relevant circuit sequestration and against routine empirical dose escalation for ECMO support *per se*. Between-patient variability is, however, wide, target attainment with standard maintenance dosing is suboptimal, and no fixed empirical regimen simultaneously meets the proposed efficacy and safety targets. The principal clinical implication is that isavuconazole exposure during ECMO should be individualized by therapeutic drug monitoring; a higher first loading dose may accelerate early target attainment but requires confirmation in larger prospective cohorts.

## Supplementary Material

dlag184_Supplementary_Data

## Data Availability

Anonymized individual concentration data and the complete analysis pipeline are available from the first author on reasonable request.

## Supplementary data

Figures S1 to S4 and Tables S1 to S4 are available as Supplementary data at *JAC-AMR* Online.
