# Supplementary material for: Population pharmacokinetics, target attainment and individualized dosing of isavuconazole in critically ill adults receiving extracorporeal membrane oxygenation
Source: JAC Antimicrob Resist. 2026 Aug 25;8(4):dlag184. doi: 10.1093/jacamr/dlag184 (PMC13504286; doi:10.1093/jacamr/dlag184)
Supplement: Supplementary file 1 [file dlag184_supplementary_data.docx]

**Supplementary Material**

**Population pharmacokinetics, target attainment and individualized dosing of isavuconazole in critically ill adults receiving extracorporeal membrane oxygenation**

***Hatzl S et al.***

**Supplementary Methods**

***Study design, regulatory classification and data collection***

The study was a single-centre, prospective, non-interventional pharmacokinetic study conducted at the Medical University of Graz between January 2021 and March 2023. The decision to prescribe isavuconazole, the indication and the dose administered were made by the treating intensive care physicians according to institutional standard operating procedures and current guidelines, independently of and prior to inclusion; no participant was assigned by the investigators to a therapeutic strategy. The first loading dose used in the unit changed stepwise over the study period as an institutional practice adaptation, prompted by the finding of our earlier pilot studies that the licensed loading regimen frequently fails to achieve the proposed target concentration during ECMO support. The only study-related procedure was the withdrawal of additional blood volume from an arterial catheter that was already in place for routine clinical care. The study therefore did not meet the definition of a clinical trial under Directive 2001/20/EC or Regulation (EU) No 536/2014 and required neither authorisation through the Clinical Trials Information System (CTIS) nor registration in a public clinical trials registry; it was reviewed and approved as non-interventional research by the institutional review board of the Medical University of Graz (33-062 ex 20/21). Written informed consent was obtained from all conscious participants; in unconscious patients, consent was deferred and obtained after recovery of decision-making capacity.

Clinical and laboratory variables were abstracted from the electronic patient data management system. Demographic characteristics, comorbidities, indication for isavuconazole, ECMO configuration, SOFA score and PaO2/FiO2 ratio were recorded at the time of ECMO initiation. Laboratory variables (creatinine, total bilirubin, aspartate aminotransferase, alanine aminotransferase) were recorded daily and are reported as the maximum value observed during the 168-h sampling window. Renal replacement therapy, concomitant medication with relevant CYP3A4 interaction potential, exchanges of the ECMO circuit or membrane oxygenator, adverse events and vital status were recorded prospectively. Circuit and oxygenator exchanges were recorded because drug sequestration onto a polymeric membrane may be greatest in the period immediately after a new oxygenator is placed in service.

***Sampling matrix: arterial versus venous concentrations***

All samples in the present study were drawn from an indwelling arterial catheter used for routine clinical care; no sample was obtained from a venous site or from the ECMO circuit itself. The concentration thresholds applied were derived from cohorts in which isavuconazole was measured in venous or central venous blood. Because isavuconazole is a low-extraction drug with a large volume of distribution and a terminal half-life of approximately 130 h, arterio-venous concentration differences are expected to be negligible at trough and throughout the maintenance phase, which is when all target-defining concentrations in this study were assessed. During and shortly after infusion, before distribution equilibrium is reached, arterial concentrations may transiently exceed contemporaneous venous concentrations; loading-phase peak values reported here may therefore be modestly higher than venous values obtained under the same regimen. This is acknowledged as a limitation in the main text.

***Derivation of the pharmacokinetic targets***

Three targets were applied a priori. (i) A steady-state trough of at least 1 mg/L was applied as the efficacy target because it is the concentration most consistently associated with clinical response in therapeutic drug monitoring cohorts of patients treated for invasive mould disease, and because it is the threshold used in our previous work in patients receiving ECMO. (ii) An AUC0-tau of at least 60 mg.h/L was applied as an alternative, exposure-based efficacy metric. This value corresponds to the 25th percentile of the steady-state exposure achieved by the licensed regimen in the adult phase 3 programme, the benchmark subsequently adopted as the exposure target in the paediatric development programme; it represents an AUC/MIC ratio of approximately 60 at the epidemiological cut-off value of 1 mg/L for Aspergillus fumigatus; it approximates the near-maximal effect exposure identified in experimental invasive pulmonary aspergillosis and in the exposure-response analysis of the phase 3 programme, in which exposures achieved by the clinical regimen were near the plateau of the exposure-response curve; and it is the target used in the most recent population pharmacokinetic analyses in critically ill patients, which permits direct comparison of our results with those cohorts. (iii) A steady-state trough below 5 mg/L was applied as a proposed safety threshold, above which hepatic and gastrointestinal adverse events have been reported more frequently.

No exposure target has been prospectively validated for mould-active prophylaxis, which was the indication in 12 of 15 patients in this cohort, and the targets applied here are therefore extrapolated from treatment cohorts. They were applied uniformly across the cohort for three reasons: adequate mould-active exposure is the pharmacological objective in both indications; breakthrough infection during azole prophylaxis has been linked to subtherapeutic concentrations; and a common target permits comparison across indications and with published ICU populations. The resulting uncertainty is acknowledged as a limitation in the main text, and target attainment in the prophylaxis setting should be interpreted as a pharmacokinetic benchmark rather than as a validated clinical threshold.

***Safety and clinical outcome definitions***

Adverse events were recorded from the first isavuconazole dose until 7 days after the end of the sampling window. Hepatic tolerance was assessed from daily aspartate aminotransferase, alanine aminotransferase and total bilirubin values; drug-induced liver injury, and any dose reduction, interruption or discontinuation attributable to isavuconazole, were recorded. Breakthrough invasive fungal infection was defined as probable or proven invasive fungal disease according to the EORTC/MSGERC and FUNDICU criteria occurring during isavuconazole prophylaxis or within 7 days of its discontinuation. Survival was analysed from ECMO initiation, and median follow-up of survivors was estimated by reverse Kaplan-Meier.

***Prior parameter values and non-compartmental details***

The literature-informed two-compartment prior used for Bayesian estimation and for the literature-reference simulation scenario had a population clearance of 4.4 L/h with between-subject coefficient of variation 38%, a central volume of 95 L with coefficient of variation 32%, an intercompartmental clearance of 6.4 L/h and a peripheral volume of 220 L with coefficient of variation 25%, with allometric scaling on body weight (exponents 0.75 for clearances and 1.0 for volumes; reference weight 70 kg). Prior central tendencies were chosen to span the range reported across published critically ill and pooled ICU or healthy-volunteer isavuconazole population models (Desai et al. 2016: clearance 2.36 L/h, central volume 49 L, intercompartmental clearance 26.6 L/h, peripheral volume approximately 460 L; Jansen et al. 2023: ICU population, total isavuconazole exposure consistent with an apparent clearance of approximately 4 L/h).

Loading-phase AUC was calculated by the linear trapezoidal rule over 0-24 h, with linear back-extrapolation from the first observed concentration at 2 h to C(0) = 0 to account for the rising phase before the first sample. Steady-state AUC0-tau was estimated over the last fully sampled dosing interval (144-168 h) and apparent clearance as dose divided by AUC0-tau at steady state. Terminal half-life was not estimated because the elimination half-life of isavuconazole (approximately 130 h) exceeded the sampling window.

***Statistical analysis***

Continuous variables are reported as median with interquartile range and categorical variables as counts and percentages. Comparisons across the three loading-dose strata were performed with the Kruskal-Wallis test; where the overall test indicated a difference, pairwise comparisons were performed with the Mann-Whitney U test and are reported as exploratory. Categorical variables were compared using Fisher exact or chi-squared tests as appropriate. Bayesian posthoc clearance was additionally compared between veno-venous and other ECMO configurations using the Mann-Whitney U test; given group sizes of 12 versus 3, this comparison is descriptive only. Survival curves were stratified by loading-dose group, by attainment of AUC0-tau of at least 60 mg.h/L, and by tertile of Bayesian posthoc clearance, and were compared by log-rank test. Univariable Cox proportional hazards regression was performed for prespecified predictors; multivariable models were not fitted given the modest event count, and all survival findings are presented as hypothesis-generating. A two-sided alpha of 0.05 was used without correction for multiple comparisons, and all p values reported in the main text correspond to those given in Table 2.

***Software environment***

All analyses were performed in R version 4.5.0 (R Foundation for Statistical Computing, Vienna, Austria) on Windows 11. Population PK and Bayesian analyses used the following packages: PKNCA v0.10 (non-compartmental analysis), mrgsolve v1.x (ODE simulation), mapbayr v0.x (maximum a posteriori Bayesian estimation), nlmixr2 v3.x with rxode2 backend (SAEM population PK fitting), survival and survminer (Kaplan–Meier and Cox regression), and tidyverse for data handling. Independent cross-validation of NCA results was performed in Python 3.x with NumPy, SciPy and pandas.

***Data preparation***

Isavuconazole concentration data and patient covariates were exported from a REDCap electronic case report form. The dataset was structured in NONMEM long format with one row per dose or observation event. The dosing schedule was reconstructed as: dose 1 (LD: 200, 300 or 400 mg) at t = 0; doses 2–6 at t = 8, 16, 24, 32, 40 h (200 mg q8h); maintenance doses from t = 52 h at 200 mg q24h. Each infusion was modelled with a 60-minute duration (RATE = AMT mg/h).

***PKNCA non-compartmental analysis***

Two analysis intervals were specified in PKNCAdata: a loading interval (0–24 h) and a steady-state interval (144–168 h). *Cmax*, *Cmin*, AUClast (linear trapezoidal) and apparent clearance (in the SS interval only) were computed. Half-life and lambda-z were not computed because terminal-phase data beyond 168 h were not available. Cross-validation in Python used numpy.trapezoid for trapezoidal AUC computation, with results agreeing to within 5% of the PKNCA values for all comparable parameters.

***Bayesian estimation***

The mrgsolve model implemented the Jansen 2023 two-compartment structure with allometric scaling.^1^ ETAs were declared in $PARAM (mapbayr requirement); $OMEGA provided the prior variance-covariance. The captured concentration was named CP (rather than IPRED, which is reserved by mapbayr for posthoc-predicted concentrations). Maximum a posteriori estimation of individual ETAs was performed by mapbayest() with default optimization settings. Goodness of fit was assessed by the observed-versus-individual-predicted plot on a log-log scale (**Figure S1**).

***Monte Carlo simulation***

Two independent simulation scenarios were performed in 5,000 virtual subjects. Scenario 1 (literature reference) used the literature informed prior with weights resampled from the ECMO cohort distribution; events were applied uniformly via mrgsolve's ev() and idata_set(). Maintenance dosing was extended to a 20-day horizon to ensure that the steady-state sampling window (336–360 h) captured a full dosing interval for all regimens (q8h, q12h and q24h). Scenario 2 (observed cohort) sampled CL and V1 from log-normal distributions fitted to the cohort's Bayesian posthoc estimates and used analytical 1-compartment steady-state formulas (justified by the long elimination half-life of isavuconazole).

***Sensitivity analysis: de novo population PK fit***

A sensitivity analysis fit a two-compartment model with allometric scaling de novo to the cohort data using the SAEM algorithm in nlmixr2 (200 burn-in, 300 EM iterations). Population estimates were tvCL = 2.4 L/h (95% CI 1.0–5.6), tvV1 = 125 L (43.4–360), tvQ = 6.0 L/h, tvV2 = 357 L (247–514). Between-subject variability was very high (CV ~150% on CL); shrinkage exceeded 80% on V1, indicating that the data could not support de novo estimation of distribution-phase parameters with adequate precision (**Supplementary Table S1**).

**Supplementary Table S1**

| **Patient** | **LD group** | **Weight (kg)** | **Age** | **ECMO mode** | **CL (L/h)** | **V1 (L)** |
| --- | --- | --- | --- | --- | --- | --- |
| P01 | 200 mg | 111.4 | 44 | vv | 4.06 | 162 |
| P02 | 200 mg | 96.1 | 60 | vv | 1.81 | 472 |
| P03 | 200 mg | 91.3 | 64 | vv | 2.48 | 141 |
| P04 | 200 mg | 103.9 | 55 | vv | 0.77 | 366 |
| P05 | 200 mg | 84.8 | 64 | vv | 14.47 | 134 |
| P06 | 200 mg | 62.1 | 59 | va | 14.69 | 136 |
| P07 | 200 mg | 82.4 | 45 | vv | 1.64 | 235 |
| P08 | 300 mg | 65.1 | 72 | va | 5.42 | 135 |
| P09 | 300 mg | 65.1 | 67 | vv | 2.01 | 294 |
| P10 | 300 mg | 59.9 | 31 | vv | 15.73 | 101 |
| P11 | 400 mg | 84.2 | 63 | vv | 13.71 | 218 |
| P12 | 400 mg | 100.1 | 64 | vv | 7.99 | 411 |
| P13 | 400 mg | 64.6 | 62 | vv | 4.09 | 270 |
| P14 | 400 mg | 57.2 | 47 | vav | 2.32 | 281 |
| P15 | 400 mg | 57.8 | 63 | vv | 2.27 | 98 |

**Supplementary Table S1.** Individual Bayesian posthoc pharmacokinetic parameter estimates against the literature informed prior, and population estimates from the de novo SAEM sensitivity fit.

Each row shows one patient. CL = apparent total clearance; V1 = volume of the central compartment. Estimates were derived by maximum a posteriori Bayesian estimation in mapbayr against the published two-compartment isavuconazole population model of Jansen *et al.* (2023; reference 13), with allometric scaling on body weight (exponents 0.75 for clearances, 1.0 for volumes; reference weight 70 kg). Cohort summary statistics were calculated on the individual posthoc estimates: median CL = 4.06 L/h [IQR 2.14–10.85, range 0.77–15.73]; median V1 = 218 L [IQR 135–288]. The log-normal coefficient of variation on CL was 116%, substantially exceeding the prior between-subject variability of CV ≈ 38%, indicating wider individual heterogeneity in the ECMO cohort than in published non-ECMO ICU populations. The de novo SAEM population fit (nlmixr2) yielded population estimates of tvCL = 2.4 L/h (95% CI 1.0–5.6), tvV1 = 125 L (43.4–360), tvQ = 6.0 L/h, tvV2 = 357 L (247–514). Between-subject variability was very high (CV ≈ 150% on CL); shrinkage was 17% on CL and 85% on V1, indicating that the data could not adequately support de novo estimation of distribution-phase parameters and that the de novo fit should be interpreted as supportive only. ECMO modes: vv = veno-venous; va = veno-arterial; vav = veno-arterial-venous. Bayesian posthoc clearance did not differ appreciably between ECMO configurations: median 3.27 L/h [IQR 1.96–9.42] for veno-venous support (n = 12) versus 10.06 L/h for veno-arterial support (n = 2) and 2.32 L/h for the single patient on veno-arterio-venous support (p = 0.45, Mann–Whitney U test, veno-venous versus all other configurations); given the group sizes this comparison is descriptive only.

**Supplementary Table S2**

| **Regimen** | **Median AUC₀–₂₄ (mg·h/L)** | **Median Cmin (mg/L)** | **AUC₀–₂₄ ≥ 60 (%)** | **Cmin ≥ 1mg/L (%)** | **Cmin < 5mg/L (%)** | **Joint efficacy + safety (%)** |
| --- | --- | --- | --- | --- | --- | --- |
| ***(A) Simulation under the literature-informed prior*** | | | | | | |
| 200 mg q24h (current)* | 42.6 | 1.20 | 20.2 | 63.2 | 99.8 | 20.0 |
| 300 mg q24h | 63.8 | 1.79 | 56.4 | 84.8 | 98.0 | 54.4 |
| 400 mg q24h | 85.0 | 2.39 | 80.6 | 92.7 | 92.2 | 72.8 |
| 200 mg q12h | 85.0 | 2.81 | 80.6 | 96.9 | 88.9 | 69.5 |
| 300 mg q12h | 127.5 | 4.22 | 96.1 | 99.4 | 63.9 | 60.0 |
| ***(B) Simulation under the observed cohort CL/V1 distribution*** | | | | | | |
| 200 mg q24h (current)* | 48.5 | 1.86 | 41.1 | 71.6 | 83.7 | 24.8 |
| 300 mg q24h | 72.9 | 2.78 | 58.4 | 82.3 | 72.3 | 30.7 |
| 400 mg q24h | 94.3 | 3.60 | 67.8 | 87.1 | 62.3 | 30.1 |
| 200 mg q12h | 95.1 | 3.78 | 68.6 | 90.1 | 60.9 | 29.5 |
| 300 mg q12h | 145.0 | 5.78 | 81.8 | 95.1 | 44.4 | 26.3 |

**Supplementary Table S2.** Probability of target attainment under five candidate maintenance regimens, derived from Monte Carlo simulation of 5,000 virtual subjects per regimen.

All regimens were preceded by the standard 200 mg q8h × 6-dose loading regimen. **(A)** Simulation under a literature-informed two-compartment prior (population CL = 4.4 L/h with between-subject CV 38%, V1 = 95 L with CV 32%, Q = 6.4 L/h, V2 = 220 L with CV 25%; allometric scaling on body weight, reference 70 kg), with weights resampled from the cohort distribution. This scenario provides the literature reference projection. **(B)** Simulation under log-normal distributions of clearance (CL) and central volume (V1) fitted to the cohort's Bayesian posthoc estimates; this scenario provides the projection most relevant to a population resembling the cohort, since it preserves the observed wider between-patient variability (CV 116% on CL versus CV 38% in the literature-informed prior). The 80% probability of target attainment is the conventional threshold for dose acceptability. The exposure threshold is daily AUC (AUC₀–₂₄ ≥ 60 mg·h/L) which corresponds to AUC₀–τ ≥ 60 for q24h regimens and AUC₀–τ ≥ 30 (per 12-h interval) for q12h regimens, equivalent to the same daily exposure target adopted by Jansen *et al.*.^1^ The joint endpoint is the simultaneous attainment of AUC₀–₂₄ ≥ 60 mg·h/L (efficacy) and proposed Cmin < 5 mg/L (safety). In the observed-cohort scenario, no fixed regimen achieves the joint target at ≥ 80% probability, supporting the suggestion of therapeutic drug monitoring–guided individualized dosing.

* current refers to current recommendantion of 200mg q24h maintenance dose

**Supplementary Table S3**

*Univariable Cox regression: predictors of all-cause mortality from ECMO start*

| **Variable** | ***β*** | **HR (95% CI)** | ***p*** |
| --- | --- | --- | --- |
| Loading dose 400 mg vs 200/300 mg | 1.27 | 2.65 (0.46–15.29) | 0.27 |
| AUC₀–τ ≥60 vs <60 mg·h/L (steady state) | 1.21 | 3.35 (0.34–33.36) | 0.30 |
| Indication: treatment vs prophylaxis | 5.66 | 14.99 (1.53–147.04) | 0.020 |
| Age (per 10 years) | 5.39 | 5.87 (1.08–31.92) | 0.040 |
| SOFA score (per 1 point) | 0.24 | 1.27 (0.98–1.66) | 0.073 |
| Bayesian CL (per 1 L/h) | 0.03 | 1.03 (0.92–1.16) | 0.61 |

**Supplementary Table S3.** Univariable Cox proportional hazards regression for predictors of all-cause mortality from ECMO initiation.

Hazard ratios (HR) and 95% confidence intervals are presented for each predictor as the only term in the model; multivariable models were not constructed given the limited event count (8 deaths in 15 patients). β = log-transformed hazard ratio coefficient; HR (95% CI) = exponentiated coefficient with profile-likelihood confidence interval; p = Wald test p-value. The associations of treatment versus prophylaxis (HR 14.99, 95% CI 1.53–147.04, p = 0.020) and increasing age (HR 5.87 per 10 years, 95% CI 1.08–31.92, p = 0.040) with mortality reflect well-established prognostic factors for invasive pulmonary aspergillosis in critically ill patients rather than a direct exposure–outcome relationship. Loading dose, AUC target attainment, SOFA score and Bayesian posthoc clearance were not significantly associated with mortality. The wide confidence intervals across all predictors reflect the small event count and underpowered nature of the analysis. Of note, none of the seven patients who received a first loading dose of 200 mg was treated for probable invasive pulmonary aspergillosis, whereas the three patients treated for probable invasive pulmonary aspergillosis received a first dose of 300 mg (n = 2) or 400 mg (n = 1). Indication and loading dose are therefore confounded, and no direct comparison of outcomes by loading dose is possible.

**Supplementary Table S4**

| **Patient** | **First dose** | **Samples 0–24 h** | **Cmax 0–24 h (mg/L)** | **Time to first ≥1 mg/L (h)** | **C at 24 h (mg/L)** |
| --- | --- | --- | --- | --- | --- |
| P01 | 200 mg | 6 | 1.71 | 2 | 1.01 |
| P02 | 200 mg | 6 | 1.16 | 24 | 1.16 |
| P03 | 200 mg | 1 | 1.35 | 24 | 1.35 |
| P04 | 200 mg | 6 | 0.90 | not within 24 h | 0.90 |
| P05 | 200 mg | 5 | 1.26 | 2 | 0.83 |
| P06 | 200 mg | 4 | 0.45 | not reached | no sample |
| P07 | 200 mg | 6 | 1.73 | 2 | 1.73 |
| P08 | 300 mg | 6 | 1.93 | 8 | 1.29 |
| P09 | 300 mg | 6 | 1.41 | 2 | 1.41 |
| P10 | 300 mg | 6 | 2.40 | 2 | 1.00 |
| P11 | 400 mg | 6 | 1.78 | 2 | 0.90 |
| P12 | 400 mg | 6 | 1.10 | 16 | 0.77 |
| P13 | 400 mg | 6 | 1.98 | 2 | 1.50 |
| P14 | 400 mg | 6 | 2.14 | 2 | 1.78 |
| P15 | 400 mg | 6 | 4.87 | 2 | 2.95 |

**Supplementary Table S4.** Attainment of the proposed 1 mg/L efficacy threshold during the loading phase, by patient. Cmax 0–24 h is the highest concentration observed among the samples drawn at 2, 4, 8, 12, 16 and 24 h after the first dose. Time to first concentration of at least 1 mg/L is derived from all observations, so that "not within 24 h" indicates that the threshold was first exceeded later in the sampling period, and "not reached" that it was never exceeded. The concentration at 24 h is a pre-dose trough and is therefore the first time point at which attainment can be regarded as sustained rather than transient. Transient attainment at any point during the first 24 h occurred in 5 of 7, 3 of 3 and 5 of 5 patients in the 200, 300 and 400 mg strata, whereas attainment at the 24 h trough occurred in 4 of 6 evaluable, 3 of 3 and 3 of 5 patients; one patient (P06) had no 24 h sample. Sampling density during the first 24 h varied between patients (one to six samples), so the figures for transient attainment are influenced by how frequently individual patients were sampled and should be interpreted with that in mind.

**Supplementary Figures**

**Figure S1.**

Goodness-of-fit plot for Bayesian posthoc estimation against the literature informed prior. Each point is one observed–individual-predicted concentration pair (n = 148). Axes are logarithmic. The red dashed line is the line of identity. Symmetric scatter around the line of identity indicates absence of systematic bias and supports the appropriateness of the prior for the ECMO cohort.

**Figure S2.**

Individual concentration–time profiles for all 15 patients, in a 3 × 5 grid layout, annotated with each patient's posthoc Bayesian clearance estimate. Patients with extreme high clearance (P05, P06, P10, P11) are highlighted (red dots); their profiles show characteristically low steady-state concentrations despite identical dosing

**Figure S3.** Simulated concentration–time profile of total isavuconazole over 168 hours under standard SmPC dosing (200 mg every 8 hours × 6 loading doses on days 1–2, followed by 200 mg every 24 hours from day 3) in 5 000 virtual subjects, under two assumed pharmacokinetic distributions. (A) Literature-informed prior with population clearance 4.4 L/h, central volume 95 L, intercompartmental clearance 6.4 L/h and peripheral volume 220 L, with between-subject log-normal coefficients of variation of 38%, 32% and 25% on clearance, central volume and peripheral volume respectively, and allometric scaling on body weight (reference 70 kg); this corresponds to the published critically-ill ICU population. (B) Observed-cohort distribution: log-normal clearance and central volume fitted to the 15 individual Bayesian posthoc estimates from this study (median clearance 4.06 L/h, between-subject coefficient of variation 116%; median central volume 218 L, coefficient of variation 54%). Both panels use identical dosing, identical body-weight resampling from the cohort distribution, and identical peripheral volume and intercompartmental clearance values. Solid line = population median; medium-shaded band = 90% prediction interval; light-shaded band = 95% prediction interval. Dashed horizontal line = 1 mg/L efficacy threshold. The marked widening of the prediction interval in panel B relative to panel A, together with the slower median rise during the loading phase, reflects the high-clearance phenotype observed in 4 of 15 ECMO patients (clearance 13–16 L/h) and explains why standard dosing fails to consistently achieve target concentrations in this cohort despite a population median clearance similar to non-ECMO ICU populations. Simulation was performed in mrgsolve with explicit per-subject sampling of inter-individual variability terms**.**

**Figure S4.**

Kaplan–Meier survival curves stratified by exposure metrics. (A) AUC₀–τ ≥60 vs <60 mg·h/L at steady state. (B) Tertile of Bayesian posthoc clearance (low CL = high exposure; intermediate; high CL = low exposure). Risk tables shown beneath each panel. The analysis is severely underpowered (8 events) and is presented as supportive cohort description.

**Alt text section:**

**Figure S1.** Three-panel goodness-of-fit diagnostic. Panel A: observed versus individual predicted concentrations on log-log axes with line of identity and LOESS smoother. Panels B and C: relative residuals plotted against time after first dose and against predicted concentration, with reference lines at zero and ±1. Points are coloured by loading-dose group.

**Figure S2.** Grid of fifteen small concentration-time plots, one per patient, ordered by Bayesian clearance from lowest to highest. Each panel is annotated with patient ID, loading dose group and clearance value, with a reference line at 1 mg/L.

**Figure S3**: Concentration-time curve over 168 hours from a Monte Carlo simulation of 5,000 virtual subjects on standard isavuconazole dosing. The median curve rises sharply during the loading phase, peaks around 48 hours, then declines slightly toward steady state by day 7. Two shaded bands surround the median curve, showing 90 and 95 percent prediction intervals; the bands widen during the loading peak and narrow during maintenance dosing. A dashed horizontal reference line marks the 1 milligram per liter efficacy threshold, which the median curve crosses upward within the first 24 hours.

**Figure S4.** Two-panel Kaplan-Meier plot truncated at 90 days. Panel A compares survival in patients with steady-state AUC above versus below 60 mg·h/L. Panel B compares survival across tertiles of Bayesian clearance. Each panel has a risk table beneath and an annotated log-rank p-value.

**References**

1. Jansen AME, Mertens B, Spriet I et al. Population pharmacokinetics of total and unbound isavuconazole in critically ill patients: Implications for adaptive dosing strategies. *Clin Pharmacokinet.* 2023; **62:** 1701–11.
